# A systemic analysis of Creutzfeldt Jakob disease cases in Asia

**DOI:** 10.1080/19336896.2024.2311950

**Published:** 2024-02-07

**Authors:** Urwah Rasheed, Sana Khan, Minahil Khalid, Aneeqa Noor, Saima Zafar

**Affiliations:** a Department of Biomedical Engineering and Sciences, School of Mechanical and Manufacturing Engineering, National University of Sciences and Technology, Islamabad, Pakistan; b Clinical Department of Neurology, University Medical Centre Göttingen and the German Centre for Neurodegenerative Diseases (DZNE), Robert, Germany

**Keywords:** Asia, Creutzfeldt Jakob disease, genetic CJD, genetic prion diseases, latrogenic CJD, sporadic CJD

## Abstract

Creutzfeldt Jakob Disease (CJD) is a rapidly progressive, fatal neurodegenerative disorder, also known as a subacute spongiform encephalopathy. There are three major subtypes of CJD i.e. Sporadic CJD, which occurs for reasons unbeknown to science (85% of known cases), Genetic or Familial CJD which is characterized by the presence of mutations in the human prion protein (PRNP) gene (10–15% cases) and Iatrogenic CJD that occurs via accidental transmission through medical and surgical procedures (1–2% cases). CJD cases occur globally with 1 case per one million population/year. Considerable data is available related to the incidence and prevalence of CJD in Europe and America. However, the global surveillance database is yet to include Asia even though several Asian countries have their own CJD monitoring units. sCJD is the highest among all CJD cases in Asia. China (1957) and Japan (1705) have reported more cases of sCJD than any Asian country and Hong Kong (1) has reported the least. On the other hand, gCJD is highest in Japan (370) and least in India (2). Our analysis establishes the presence of all variants of CJD across Asia. However, in most Asian countries in general and Southeast Asian countries in particular, CJD cases are misdiagnosed and often underreported. Since Asia is the most populated continent in the world, the actual global prevalence of CJD cannot be estimated until and unless these countries are accounted for. Concrete and reliable surveillance networks are needed across Asia to evaluate the prevalence and incidence of CJD in the region.

**Figure:**
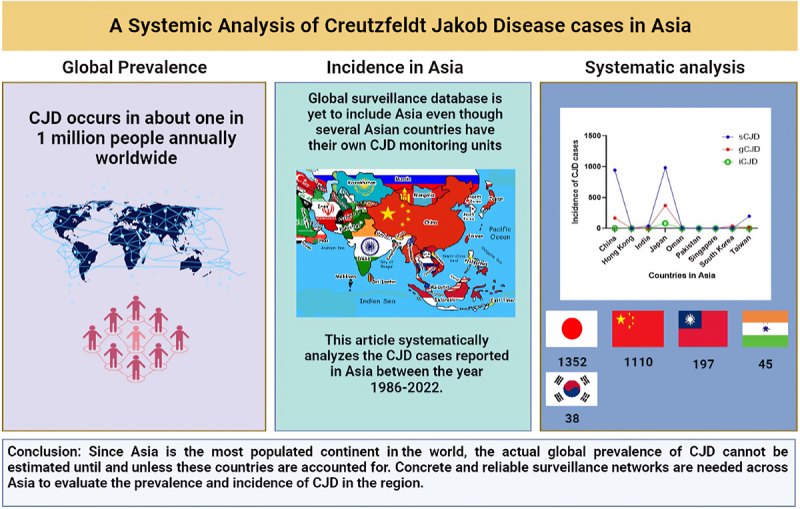

The graphical abstract demonstrates the prevalence of CJD cases in the world and systematically analyses the incidence of CJD in Asian countries between the year 1986–2022. Highest number of cases were reported in Japan followed by China. The study emphasizes the need for assimilation of Asian data in global prevalence.

## 1. Introduction

Prion disease is a rare, lethal neurodegenerative disease that affects humans and animals alike. It is caused bymisfolding of prion proteins (PrP). It was previously called Transmissible Spongiform Encephalopathy and the term Prion was derived from proteinaceous infectious particle by Stanley B. Prusiner in 1982. Although prion diseases in humans are found to be extremely rare (two per million), Creutzfeldt Jakob disease (CJD), a type of prion disease, is the most common among them worldwide [1]. It was first explained in 1920 by German neurologist Hans Gerhard Creutzfeldt and later by Alfons Maria Jakob, after whom the name was devised [2].

A normal cellular prion protein (PrP^C^) is a glycophosphatidylinositol-anchored glycoprotein with a largely α-helical C-terminal domain and an intrinsically disordered N-terminal domain that binds with copper (*Access is deniedAccess is denied* and zinc (*Access is denied Access is denied*) ions. Usually found on the cell surface, PrP^C^ can also be situated on the luminal side of intracellular bodies. It is expressed in various cell types both in the central and peripheral nervous system, but it is largely found in the neurons [3]. The synthesis of this protein is carried out in the endoplasmic reticulum and Golgi apparatus as glycoproteins bound to the cellular membrane by a glycophosphatidylinositol anchor. PrP^C^ molecules travel to the cell surface through the secretory pathway where they are exposed. In most cases, the diseased protein carries β-sheets and loops assembled into multimers such as amyloid fibrils, instead of the α-helical structure found in a normal individual [4].

While describing the physiological role of the PrP^C^ protein in healthy individuals has been complicated, the fact that misfolding of this protein generates pathological proteoforms in prion diseases is clear. Studies have been conducted by differentially regulating the levels of the protein to assess their influence on cellular functions. The changes manifested as altered phenotypes, which include impairments in development, homoeostasis, circadian rhythm, stress responses, and synaptic plasticity [5]. Another reported role of PrP^C^ protein is the mediation of neurotoxic effects in Alzheimer’s disease models caused by amyloid–β oligomers. Based on this study, the functions carried out by the PrP^C^ protein have been determined to be homoeostasis (AccessisdeniedAccessisdenied and $Zn^{2+}$ binding), cellular signalling, ion channel regulation, modulating neuronal excitability via NMDA receptor, cell adhesion (neurite outgrowth), maintaining peripheral nerve myelin, neuronal survival and differentiation, protection from reactive oxygen, and as a receptor for amyloid-β oligomers in Alzheimer disease [6].

**Figure 1.**
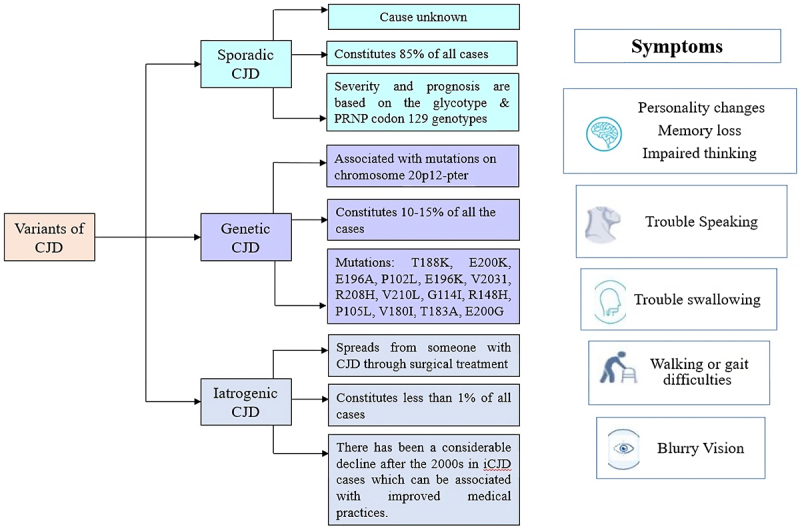
Variants of CJD and symptoms: the figure briefly describes the variants of CJD – sporadic, genetic, and iatrogenic along with their generalized symptoms.

CJD occurs in three forms: sporadic, familial, and, iatrogenic or acquired (Figure 1). Majorly about 85% of cases turn out to be sporadic (sCJD), whereas about 10%−15% of the cases are genetic (gCJD) and less than 5% of the cases are iatrogenic in nature. Very few cases are acquired through infection when exposed to Bovine Spongiform Encephalopathy (BSE). The number of cases of acquired CJD saw a sharp decline when mishaps in treatment were reduced with time [7–9].

### 1.1. Sporadic CJD

On average, the age of onset of sCJD has been observed to be 70 years [9]. The disease is recognized mainly by visual symptoms, cerebellar ataxia, rapidly progressive cognitive decline, pyramidal and extrapyramidal signs, and myoclonus. Apart from these, some psychiatric symptoms also appear, such as insomnia and behavioural changes [10]. Depending on the age, gender, $\mathrm{Pr}P^{\mathrm{Sc}}$ (diseased protein derived from ‘Scrapie’) glycotype and genotype of the gene prion protein codon 129, the disease effects differently. The survival rate depends on the age of onset of CJD in patients. In a patient above the age of 80, the period between the diagnosis and death is very short (3 months), while those around the age of 50 have a mean survival of 7 months. Moreover, female patients have been known to live a month longer than male patients affected by CJD. The disease severity, susceptibility, and prognosis are based on the $\mathrm{Pr}P^{\mathrm{Sc}}$ glycotype and PRNP codon 129 genotypes [11].

#### 1.1.1. PRNP codon 129 genotypes

The genotype of the PRNP codon 129 affects the period of survival of a patient. Studies have classified the genotypes as methionine (M) and valine (V), which have further six subtypes: MM1/MV1, MM2 cortical, MM2 thalamic, MV2, VV1, and VV2. Approximately 50% of the general population of European countries carry heterozygosity (MV) for codon 129 [12,13]. Around 80% of patients with sCJD are found to carry either homozygous methionine or valine, which shortens the lifespan of diseased patients in comparison to heterozygous patients [11,14].

#### 1.1.2. PrP^CJD^ Glycotype

There are 2 glycotypes found to be accumulating in the brain in CJD i.e., Type 1 and type 2A. The distinguishing feature of the two types is the electrophoretic migration of the unglycosylated fragments, i.e., the unglycosylated $\mathrm{Pr}P^{\mathrm{Sc}}$type 1 fragment migrates at 19 kDa, while type 2A migrates at 21kDa. Each glycotype prefers to associate with a specific polymorphic codon 129 genotypes. Type 1 has a high tendency of combining with the MM genotype whereas type 2 tends to be inclined towards VV and MV genotypes [11,14].

### 1.2. Genetic CJD (gCJD)

gCJD, also known as familial CJD (fCJD), is a rapidly progressive, fatal neurodegenerative disorder characterized by mutations in the human prion protein gene (PRNP) [15]. In most people diagnosed with CJD, no identifiable cause is known, however, 10–15% of all CJD cases are genetic with either point mutation or insertion of octapeptide repeats in the human PRNP gene [15,16]. The inheritance pattern of this gene is autosomal-dominant with variable penetrance which means that only one mutated copy of the PrP gene is enough to cause the disease [17].

### 1.3. Iatrogenic CJD (iCJD)

CJD can also be acquired during a process of medical treatment or from contaminated medical devices and materials used for transplantation. One of the first cases reported in the 1990s was that of a patient diagnosed with CJD acquired after corneal transplantation [18]. Eventually, a larger number of cases were being reported indicating an outbreak of CJD in patients with contaminated growth hormone and dura mater grafts. The onset of iCJD symptoms starts between 2 and 18 years after transplantation surgery. The symptoms which occur as a result of contamination occurring near the brain are similar to those exhibited in sCJD i.e., rapidly progressive dementia, myoclonus, and ataxia. Studies depict that the younger population has a higher chance of contracting iCJD [19]. Fortunately, there has been a considerable decline after the 2000s in iCJD cases which can be associated with improved medical practices with better infectivity control and the use of recombinant growth hormone and autologous or synthetic dura matter grafts [20].

### 1.4. Clinical characteristics of CJD

The clinical course of typical CJD cases can be divided into three stages.

#### 1.4.1. First stage

Patients in the age group between the 50s and 60s have depicted a higher incidence rate of disease, although recent cases show later onset is emerging. Initially, the symptoms appear as dizziness, fatigue, decrease in activity, disturbed memory, visual disorder, and psychological issues such as depression and anxiety. Prior to the clinical onset of the disease, the cerebral cortex and striatum appear as hyper-intensive regions in Diffusion-Weighted Magnetic Resonance Imaging (DWI MRI).

#### 1.4.2. Second stage

Cognitive dysfunction deteriorates quickly. Along with the appearance of myoclonus, difficulty in communication with progressing gait disturbances and walking difficulty, render the patient bedridden. Moreover, neuropsychiatric signs, such as apraxia, aphasia, sensory disturbance, motor paralysis, cerebellar ataxia, dystonia, startled reaction, muscle rigidity, and pathological reflex, begin to appear. Electroencephalography (EEG) testing depicts Periodic sharp-wave complexes (PSWCs).

#### 1.4.3. Third stage

In this stage, a state of akinetic mutism occurs with the patient presenting tetraplegia or abnormal posture rigidity in flexion along with contracture. The myoclonus, the hyperintensity regions which appeared on DWI, and PSWCs which appeared on EEG reduce with time and eventually disappear. The patient then dies due to infection, respiratory failure, aspiration pneumonia, or general prostration [2].

### 1.5. Diagnosis

In the 1960s, CJD was typically diagnosed on the basis of EEG findings and changes in the neuropil. With the development of modern techniques, certain diagnostic criteria have been set to confirm CJD [8]. Early diagnosis of CJD is a challenge yet to be overcome due to the variable and non-specific clinical onset of the disease. Rapidly progressing dementia characterized by diminished multiple cognitive domains can be diagnosed as sCJD only after crossing out metabolic diseases (such as heavy metal toxicity or Wernicke encephalopathy), neoplastic, paraneoplastic, and autoimmune diseases. To derive to the conclusion as CJD during diagnosis, $\mathrm{Pr}P^{\mathrm{Sc}}$ should be positive. In case of its absence, supporting tests (such as detecting PSWCs on EEG, diffusion patterns on MRI, and 1433 protein in the cerebrospinal fluid (CSF) can be done to aid in the diagnostic process, albeit they cannot be used for definitive diagnosis alone [21]. In recent years, however, significant developments have been in the diagnostic processes. Studies suggest that ultrasensitive seeding assays based on $\mathrm{Pr}P^{\mathrm{Sc}}$ amplification can stipulate a basis for an accurate antemortem diagnosis of prion diseases, reduction of iatrogenic prion transmission, and biomarker-based evaluation for future therapeutic trials [8].

## 2. Methodology

Utilizing the PubMed database, the search encompassed the time frame from 1986 to 2023 for CJD cases in Asia. No restrictions were imposed on language or publication dates. The study focuses on the incidence of the three main types of CJD and other phenotypes of genetic prion diseases in the population and was conducted using the keywords *Creutzfeldt-Jakob Disease, CJD incidence, sporadic CJD, genetic CJD, iatrogenic CJD* and *genetic prion diseases*. The systematic review adhered to the subsequent criteria for inclusion and exclusion:

Inclusion Criteria:

- The case studies belong specifically to Asian countries.
- The patient must present no copathologies.

Exclusion Criteria:

- The case studies belonging to countries other than Asian countries.
- Case studies which do not mention age, gender and diagnosis criteria.

Three reviewers independently evaluated the titles and abstracts of all the papers identified through the search based on the criteria for inclusion and exclusion, resolving any inconsistencies in the final lists of studies included through deliberation. Subsequently, potentially relevant articles were identified and cross-referenced with the lists for inclusion and exclusion.

## 3. Global incidence of creutzfeldt Jakob Disease

Annually, the European Center for Disease and Prevention control reports the incidence of CJD globally. Their recent census depicted a low rate of deaths from 1996 to 2018 in iCJD and gCJD whereas an apparent steady increase in sCJD cases [22]. Low incidence of iCJD and gCJD cases can be attributed to increasing awareness whereas a higher incidence of sCJD supports the idea of a global ageing population [23]. In contrast, this global surveillance database is yet to include Asia even though few Asian countries like China, Taiwan, and Japan have their own CJD monitoring units.

## 4. Incidence of sporadic CJD in Asia

### 4.1. China

China recorded the highest number of sCJD cases since the early cases emerged in 1982. Guo et al., published two case reports, out of which one was sporadic, whereas the other was genetic. While the source of infection for the sporadic case could not be identified, the EEG showed progressing changes aiding in the clinical diagnosis [24]. Following the initial reports, the Department of Neurology, General Hospital of the People’s Liberation Army diagnosed 57 cases of sCJD, between the years 1992 and 2011. Out of these 57, 11 patients were diagnosed with sCJD, 39 were probable and 7 were possible diagnoses. Of these cases, 24 were females and 33 were males, with a gender ratio of 0.727:1. The onset age range is 36 to 75 years with 8.8% of the patients in the age group 30 to 39, 14% of the patients were in 40–49 years, 35.1% were in 50–59, 36.8% were in 60–69 and 5.3% were in above 70 age group. A follow-up study was conducted, but most patients were deceased. The time between the onset of disease and death ranged from 5 to 22 months. Among the deceased, 28 died 7 to 12 months following the onset of the disease, 17 died after a year of onset, and only 9 lived up to 6 months of onset [25]. In another report published by Shi et al., 192 suspected cases of CJD were identified between the years 2006 and 2007 through surveillance systems. Fifty-one patients out of these were diagnosed as probable sCJD cases, while 30 of them were considered as possible sCJD cases, on the basis of diagnosis criteria [26].

With an increase in the elderly population, more cases have emerged recently. In 2016, Shi et al., reported the surveillance data were retrieved from 15 hospitals and 12 provincial CDCs which covered Shanghai, Beijing, Chongqing, Tianjin, Hubei, Jilin, Shaanxi, Guizhou, Guangdong, Henan, Anhui, and Xinjiang. A total of 1585 cases were reported, out of which 700 were categorized as sCJD. Two out of these were definite, 560 were probable and 138 were possible [27]. Overall, 5078 CJD cases have been reported between the years 2006 to 2021, out of which 1900 cases were attributed to sCJD cases. All PLADs located on the Chinese mainland experienced cases of sCJD, with the sole exception being the Xizang Autonomous Region (Tibet). It should be noted that there was no discernible correlation between the occurrence of sCJD and factors such as geographical location, season, or occupation.

The initial indications and manifestations of sCJD cases exhibited a wide range of diversity. The prevailing symptom that was frequently cited was the gradual onset of dementia, accounting for approximately 41% of the reported cases. This was followed by impairments in cerebellar and visual functions, which constituted 18% of the cases, mental health issues at 13%, and pyramidal and extrapyramidal symptoms at 10%. As the condition progressed, a greater number of neurological abnormalities became evident. It is worth noting that all sCJD cases were accompanied by dementia, as indicated by the reports. Furthermore, the other four symptoms associated with sCJD, namely visual or cerebellar dysfunction (67%), myoclonus (76%), pyramidal or extrapyramidal symptoms (80%), and mutism (39%), were also frequently observed. A total of 19%, 40%, and 41% of the patients displayed dementia along with 4, 3, and 2 additional neurological symptoms, respectively. Small granules and the type-1 PrPSc molecule exhibited extensive dispersion throughout the cerebral tissues of a limited cohort of individuals affected by sCJD subsequent to neuropathological and molecular evaluation, whether conducted posthumously or through cerebral biopsy [28].

### 4.2. Hong Kong

A case report of a 67-year-old was published by Chan et al., in 1987. The patient was suffering from the clinical onset of a disease for the past 6 months and was admitted to the hospital with a worsening mental state. Apart from the physical symptoms, EEG depicted irregular activities in all areas of the brain. Although the patient was reactive to painful stimuli, slight droopiness in the left central face was observed, with myoclonic jerks occurring. He was then diagnosed with CJD. The patient then slipped into coma for the next 2 months with myoclonic jerks slowly disappearing. The patient was declared deceased 4 months following his admission. His survival period from onset to death was 10 months [29].

### 4.3. India

India is a developing country, and not many cases were reported. One of the reasons could be the lack of applicable diagnostic techniques. In the period between 1971 and 1990, 30 patients were documented which included 20 confirmed and 10 probable cases of CJD. Among them, 18 were men and 12 were women. The age of onset ranged between 34 and 76 years. The survival time ranged from 1 month to 36 months. The patients had clinical onset with psychiatric symptoms such as visual complaints, and confusion state before they were diagnosed with CJD [30,31].

In another case reported by Biswas et al., 10 people were categorized as probable sCJD in the year range 2011–2013 with a gender ratio of M: F = 4:6. Their ages ranged from 39 to 70 years and averaged 56.1 years. Six of them presented clinical characteristics such as behavioural abnormalities, four of them had ataxia, five presented extrapyramidal features, four complained of visual hallucination and one had cortical blindness. Within 4 months, all of them were rendered bedridden and eight died within a mean duration of 4.56 months following onset. The remaining two patients were not available for follow-up [32].

### 4.4. Japan

After China, Japan recorded the highest number of CJD cases. From 1999 to 2009, the CJD Surveillance Committee of Japan registered 1,241 cases of prion diseases. Among them, 76.8% were sCJD cases, found mainly of MM2 genotype in thalamic and cortical forms [33]. Furthermore, Iwasaki et al., reported 29 autopsied sCJD patients recorded by the Institute for Medical Science of Aging, Aichi Medical University from the year 1997 to 2009. The patients were found to be carrying the MM1 genotype of PRNP codon 129. The mean age at disease onset was 67.1 years and the mean survival time was 11.9 months. In order to diagnose, EEG was done after the onset of symptoms. Once the akinetic mutism state was reached, the period of progression to death was 9 months [34].

A 2022 study by Kosami et al., reported 2440 CJD cases with sCJD attributed for 1676 (69%) cases. Patients with homozygosity for methionine may potentially face an elevated susceptibility to the development of prion disorders. The odds ratio (95% confidence interval) adjusted for sCJD was calculated to be 2.21 (1.46 to 3.34) [35]. In 2023, a 72-year-old female patient arrived at the medical facility displaying visual impairment in both eyes and a duration of light sensitivity spanning two to 3 months. Her visual acuity in both eyes was recorded as 20/2000 seven days prior. Upon examination, her pupillary light reflex remained intact and fundoscopy revealed no abnormalities. However, an observation of left homonymous hemianopia and limited downward movement of the left eye was possible. Upon admission, her visual acuity was reduced to light perception. Both electroencephalography and cranial magnetic resonance imaging results exhibited no irregularities or periodic synchronous discharges. On the sixth day of hospitalization, an examination of the patient’s cerebral spinal fluid exposed the presence of tau and 14-3-3 protein, with a successful real-time quaking-induced conversion outcome. Subsequently, the patient experienced myoclonus, became mute, and eventually succumbed. Postmortem analysis revealed spongiform alteration and thinning of the right occipital lobe cerebral cortex. Deposits of irregular PrP and enlarged astrocytes were observed with immunostaining. Through the examination of brain tissue via western blot and the analysis of the PrP gene’s codon 129 polymorphism, it was subsequently established that she possessed the Heidenhain variant of sCJD characterized by the presence of both methionine/methionine type 1 and type 2 cortical form [36].

### 4.5. Oman

Two sporadic cases were reported during the period between 1991 and 1995, in Oman [37]. Aged 50 and 75, both presented clinical characteristics such as rapidly progressive dementia and myoclonic jerks. The patients were diagnosed based on the symptoms and EEG results depicting abnormalities.

### 4.6. Pakistan

Pakistan reported 2 probable sporadic cases in the year 2012 and 2013. The 62- and 72-years old females were diagnosed with sCJD after both presented psychiatric and behavioural symptoms along with positive EEG and MRI results. Both patients had no history of hormone therapy or grafting [38]. Furthermore, one case of CJD was confirmed through biopsy [39]. This 69-year-old female patient was brought into Agha Khan University Hospital, Karachi, Pakistan, with behavioural symptoms which began 5 months earlier. She was also suffering from falls and gait unsteadiness for 2 months before admission. Weeks following admission, the patient started facing myoclonus jerks, eventually leading to unresponsiveness, and was rendered bed bound. To confirm a diagnosis, multiple tests were performed. A biopsy of the frontal lobe of the brain was done which depicted spongiform changes. The biopsy sample was immune stained with 3F4 monoclonal antibody, revealing granular deposits that are specifically found in prion diseases [40].

### 4.7. Singapore

Two sCJD cases, a 50-year-old male, and a 61-year-old female, have been reported in the years 1993 and 2002, respectively. The former was admitted to the hospital due to a deteriorating mental state. He presented behavioural changes, poor coordination, and jerky movements. An EEG was performed after several other tests, which demonstrated superimposed periodic high-voltage, sharp, and slow wave complexes in both hemispheres. A biopsy was also done from the frontal right lobe which showed degeneration of the grey matter in the cortical area, along with astrocytosis. The patient died a year later. The second patient’s EEG showed periodic lateralized epileptiform discharges (PLEDs) in the brain’s left frontal and central regions. After the confirmed diagnosis, the patient was bedridden for 10 months after onset [41].

### 4.8. Taiwan

Taiwan CJD Surveillance Unit (CJDSU) was established in 1997 and registered CJD cases dating back to 1975. Prior to 1997, 74 cases of sCJD were reported, with 3 biopsy-proved cases only. A total of 809 cases were reported to the CJD surveillance unit from 1996 to 2020 for the purpose of confirmation. Among these cases, 441 (with 230 women) were determined to be sporadic CJD. The average age of onset was 67 years with a standard deviation of 9.9 years. The median survival period was found to be 13.3 to 14.2 months on average. The two primary clinical signs observed were myoclonus (73% of cases) and akinetic mutism (54% of cases). In terms of PRNP polymorphism, 99% of patients (195 out of 197) exhibited the methionine homozygous genotype at codon 129 (M129M). EEG demonstrated sensitivity to periodic sharp wave complexes (PSWCs) in 59.7% of cases. The sensitivity of total tau protein (>1200 pg/mL) and 14-3-3 protein (>1200 pg/mL) in cerebral fluid was determined to be 75.6% and 69.7%, respectively. It was observed that patients who were younger tended to have longer survival compared to those who were over 65 years old (hazard ratio of 0.466, P .001). In the initial triennial period, the likelihood of survival for women surpassed that of men (hazard ratio [HR] 0.712, *p* = .005). The presence of periodic sharp wave complexes (PSWCs) continued to exert a deleterious influence on survival (HR 0.788, *p* < .05). Among the various factors, only epileptic seizures emerged as a clinically significant predictor for survival duration, despite their infrequency (HR 0.768, *p* < .05). PSWCs represent a prognostic EEG biomarker that can be effectively capitalized upon. Although rare, epileptic seizures serve as the sole clinical prognostic marker for a brief period of survival [42].

### 4.9. Thailand

While there are no official surveillance or statistics on CJd in Thailand, 2 probable and 1 possible case between the year 2012 and 2014 were reported from Thammasat University Hospital. A 42-year-old Thai woman exhibited an irregular walking pattern and subacute dizziness. After 2 weeks, she began experiencing cognitive decline, including memory loss and difficulty concentrating. Her level of enjoyment and interest in activities significantly diminished. Eventually, she became uncommunicative and developed rigid muscles as her overall health worsened. Subsequently, her limbs exhibited spontaneous myoclonus movements. T2-weighted and diffusion-weighted imaging revealed heightened signal intensity in the bilateral caudate nuclei, putamen, and left frontotemporal cortex. Electroencephalography detected generalized and synchronous periodic sharp wave complexes occurring at intervals of 0.6–0.8 seconds. Further analysis with back-averaging EEG confirmed multifocal myoclonus originating from the brain. Unfortunately, 32 weeks after the initial onset of symptoms, she passed away. The patient’s clinical history, physical manifestations, EEG findings, and brain MRI results all align with a probable diagnosis of sCJD.

In another case, a 76-year-old Thai male patient sought medical attention due to a three-week history of mental confusion, hallucinations, difficulties in speech articulation, and slight weakness on his left side. He denied ever experiencing a headache or a fever. The analysis of his CSF and the general blood chemistry were both consistent with acceptable values. On fluid-attenuated inversion recovery (FLAIR) MRI sequences, his bilateral caudate nuclei, putamen, and bilateral fronto-temporo-parietal cortices demonstrated symmetrical hypersignal intensity, with restricted diffusion on diffusion-weighted imaging (DWI). His electroencephalogram (EEG) detected periodic generalized sharp wave complexes. As time progressed, his clinical condition deteriorated, and he began to experience involuntary muscular contractions throughout his body. Sadly, he developed aspiration pneumonia, and after a duration of 8 months from the initial onset of symptoms, he tragically passed away. The patient’s condition was identified as being consistent with sCJD, with a high probability.

In the same period, a female Thai teacher, aged 53, who had previously suffered from exhaustion, insomnia, and vertigo, presented with symptoms. These symptoms included memory impairment, reduced motor coordination, and withdrawal from social interactions 1 month later. Upon physical examination, the patient displayed alertness but a significant decline in her ability to produce speech. We observed general stiffness and occasional spontaneous muscle contractions. Following an initial diagnosis of akinetic-rigid syndrome, comprehensive blood tests and cerebrospinal fluid investigations were conducted. A CT scan of the brain revealed no abnormalities. EEG detected intermittent delta slow waves superimposed on a characteristic alpha background. The patient’s condition rapidly deteriorated, resulting in muteness and confinement to bed. Spontaneous muscle contractions in the limbs were observed. Four months after the onset of symptoms, the patient passed away. Based on the clinical history, disease progression, and physical examination findings, a potential diagnosis of sCJD was indicated [43].

## 5. Incidence of genetic CJD in Asia

### 5.1. China

CJD was hardly diagnosed in China till the end of the 1980s. According to the data provided by Chinese National Surveillance for CJD, a total of 5,078 cases of CJD were reported of which 243 cases accounted for different types of Genetic Prion Diseases (gPRDs) including gCJD. Of all the prion disease (PrD) cases diagnosed between 2006 and 2021, 11.1% were gPrD cases of which 167 were confirmed as gCJD [44]. Overall, 19 different subtypes of PRNP mutations were identified in Chinese patients [45]. CJD cases were diagnosed according to the criteria provided by the Chinese National Health Commission and World Health Organization. Clinical examinations including MRI, EEG, CSF 14-3-3, CSF tau, and CSF biochemistry analysis was done followed by PRNP PCR and sequencing. The results were clinically correlated by the expert neurologists and epidemiologists.

The most common mutation in China is T188K with 65 cases i.e., 29.8% of all the gPRD cases. T188K cases have been identified in 20 provinces across China, with largest number of cases in Shandong. First case of T188K mutation in China was identified in 2009. Median age at the onset of gCJD with T188K mutation is 61 years and the average survival time of the patient is 4 months. MRI of 78% of the T188K patients showed special abnormalities [46,47]. Only a single instance of the eight CJD T188K cases documented in one study exhibited a positive familial background of the ailment. The affected individuals ranged in age from 39 to 76 years old. The initial symptoms are reportedly inconsistent. The predominant clinical presentation in most patients (63%) was rapidly progressive dementia, followed by walking instability (50%), in that particular sequence. Each individual experienced dementia, as well as pyramidal or extrapyramidal complications, during the later stages of the condition. Moreover, cerebellar dysfunctions and myoclonus were also frequently observed, akinetic mutism was also recorded [47]. Another study that analysed 30 patients with the T188K mutation concluded that progressive dementia observed among these patients was quite prevalent, with a prevalence rate of 66%. Additional early markers included extrapyramidal symptoms, mental dysfunction, and cerebellar problems at a rate of 30%, 33%, and 40%, respectively. Visual abnormalities and akinetic mutism were also observed in the later stages of the illness among the patients. Approximately 27.9% of T188K mutation cases showed periodic sharp wave complexes (PSWC) on EEG [48].

The second most common mutation to have been diagnosed in China is E200K with 41 cases in total and constituting 19.8% of the total gPRD cases [44,49]. Only 6 out of 41 E200K patients showed family history. Increased proportion of CSF tau protein was observed in these patients. The first Chinese case of E200K mutation was diagnosed in 2010 which displayed missense mutation in codon 200 (E200K) and methionine homozygous genotype at codon 129 of PRNP gene [50]. In a study, 30 patients with E200K mutation were analysed, showed that 14-3-3 marker was detected in a majority of the patients. The EEG pattern exhibited an anomalous nature characterized by a decrease in the rate of background activity and the presence of periodicity, accompanied by triphasic sharp waves. In nearly half (48%) of the gCJD cases pertaining to the E200K mutation, the presence of PSWC on EEG recordings were identified [48]E200K cases are more prevalent in Northern China and are identified in 16 provinces. The average age at the onset of E200K mutation gCJD is 57 years and survival time is 6 months [44].

The third most common mutation in gCJD patients is E196A with a total of 16 cases till date and 7.3% of the entire gPRDs [48,51]. None of the patients with E196A reported any family history of CJD. A total of 73% of the patients with E196A mutation displayed special abnormalities on MRI. The average age at the onset of gCJD in patients with E196A mutation is 61 years and the survival time is six and half months [44]. E196A patients show similarities with sCJD phenotypically with slight differences in a few clinical features [52]. In a study, it was reported that an elderly Chinese male, aged 76, who was diagnosed with CJD, exhibited a unique mutation in the PRNP gene. The presence of the 14-3-3 protein was detected in the cerebrospinal fluid, while diffusion-weighted MRI scans revealed the existence of a ribbon-like high signal intensity in both cortices. Furthermore, electroencephalography demonstrated a typical periodic synchronous discharge. The diagnosis of CJD was established based on the manifestation of distinct clinical symptoms. Remarkably, the identification of a point mutation at codon 196 (E196A: GAG→GCG) within the PRNP gene was made [53].

Other mutations with less than 5 cases throughout China are E196K, V2031, R208H, V210I, G114I, R148H, V180I, T183A and E200G with 5, 3, 3, 3, 2, 2, 1, 1, and 1 case identified respectively [54–57].

### 5.2. India

Due to the lack of genetic testing facilities, reports regarding gCJD from India are limited [58]. Only two studies identifying genetic or familial CJD have been carried out so far. First familial CJD case with D178N from South-East Asian region was reported in India. A 42-year-old male patient exhibited a history of dementia, behavioural problems, and difficulty in walking. Over the course of 3 months his symptoms exacerbated rapidly, and he developed myoclonus. He also had a family history on his maternal side of the family where his family members developed similar symptoms. Genetic testing revealed D178N mutation in the PRNP gene. CSF 14-3-3 turned out to be negative which is in accordance with the previous studies which discovered that CSF 14-3-3 is negative in patients with D178N mutation. Neuroimaging and clinical evaluation also indicated CJD. All the symptomatic members of his family died within 3 to 15 months of the onset of the disease. Two more family members were also tested positive for D178N mutation and V/V polymorphism was identified at 129^th^ amino acid but they were asymptomatic [59].

In another case report in India, a man presented a history of short-term memory loss, behavioural changes, mood swings, deterioration in communication, hand tremor, lost control of urination. Neuropathological analysis showed major neuronal loss along with the shrinkage of cortex. Rare small plaques were seen in PrP immunostaining. Genetic analysis showed D178N mutation in PRNP. Genetic testing on 27 members of his was performed, the results showed that two of his family members were positive for D178N mutation [60].

### 5.3. Japan

In 1996, three gCJD patients were identified with M232R mutation in Japan. The patients exhibited rapidly progressive dementia, abnormal EEG results and myoclonus. All three patients suffered akinetic mutism within 2 to 6 months of developing the disease and died within 4 to 24 months. Diffused grey matter was seen in immune PrP staining. Loss of neurons could be seen in histopathological analysis. Plaque formation in the brain wasn’t observed. Healthy controls did not have the M232R mutation indicating that the disease in those three patients was indeed caused by M232R [61].

In another report, two brothers were reported to have familial CJD, both patients presented rapidly progressive dementia, abnormal EEG, and myoclonus. One brother was 62 years old and died within 6 months of developing the disease. The second brother was 58-year-old when he developed the disease and died within 13 months. Genetic analysis identified point mutation in PrP gene at codon 200 of one of the first brother. Heterogenous phenotype of CJD200 was identified in Europe and America whereas in Japan the phenotype was homogenous [62]. In 1997, another family was reported to have familial CJD with codon 200 and codon 219 mutation [63]. In 1999, Japan established its national surveillance system to identify prion diseases in humans. This program collected clinical, neurological, and genetic information of patients suffering from CJD from 1999 to 2014. Of 2,394 cases identified, 365 were of gCJD. The overall prevalence was 1.2 cases/million per year. According to the genetic testing, the most prevalent mutation was V180I with a total of 211 cases followed by M232R and E200K with 63 and 61 cases respectively. Currently, there are numerous reports indicating that the V180I mutation in PRNP is responsible for distinct clinical and pathological findings. Due to the rarity of a familial history of the disease among patients with V180I, the question of whether this mutation is the cause of prion disease persists. Conversely, patients with V180I exhibit various specific clinical features that differentiate them from those diagnosed with Scjd or other genetic prion diseases (gPrDs). Patients with V180I can be easily distinguished from those with other forms of dementia due to the presence of specific hyperintensity observed in the cerebral cortex during diffusion-weighted MRI [64] Five cases of D179N were also identified [59]. Five cases of D179N were also identified [65].

### 5.4. South Korea

Due to the close proximity of the Republic of Korea and Japan, similar patterns of mutations were identified, however data related to mutations and polymorphisms associated with gCJD is scarce in Korea. A surveillance study was carried out from 2001 to 2019 in Korea, during this period 33 were familial or genetic. The mutations that were prevalent in South Korea include E200K, V1801, M232R, D178N, and V203I [66].

In a study, in addition to MRI and EEG, genetic analysis was carried out which revealed gCJD patients had three mutations at codon D178N, E200K, and M232R. The D178N mutation (with homozygous M/M at codon 129) was initially identified in 2009 in a male patient of age 67 who suffered from atypical CJD, without any familial background. The observed symptoms consisted of gradual disruption of gait and speech difficulties accompanied by extrapyramidal indicators, but not insomnia. Additionally, the patient displayed rigidity and bradykinesia, yet there was an absence of myoclonus, visual impairment, cognitive decline, or pyramidal symptoms. Over the course of a few months, the patient’s condition rapidly deteriorated into akinetic mutism, although the duration of the disease was relatively extended, exceeding 2 years. EEG results were within the normal range, with positive findings for the 14-3-3 CSF marker. MRI scans exhibited elevated signal intensities in both the parietal and occipital gyri [67].

The 58-year-old patient with E200K mutation displayed swift disease progression, impaired gait, cognitive disarray, and myoclonus. Subsequently, dysarthria, diminished gait, decreased attention, and restlessness manifested. During the neuropsychological assessment, the patient exhibited signs of confusion and disorientation. The patient succumbed to the illness 3 months after the initial onset of symptoms. MRI unveiled heightened signal intensity in various regions of the brain, encompassing both the bilateral frontal temporoparietal area and the caudate nucleus. EEG revealed the presence of sharp spikes and slow waves, and CSF tested positive for the 14-3-3 signal [67]. Another case of gCJD with E200k mutation was documented in Korea, the patient, displayed the onset of progressive dysarthria and visual hallucinations at the age of 63. Subsequently, within a brief period, the patient also encountered impairments in gait, myoclonus, and behavioural abnormalities. The DWI examination revealed significant elevation in signal intensity within the basal ganglia and occipitoparietal cortex regions. A patient who was 65-year-old with M232R mutation exhibited rapidly progressive dementia and walking disability, MRI displayed heightened signal intensities in the parieto-occipital cortex and temporal lobes and CSF exhibited positivity for 14-3-3 protein. The patient died within 16 months of developing the disease [67].

In another case, a 75-year-old woman was reported to have fCJD with V180I mutation. This is the first case of a point mutation at codon 180 in South Korea. The EEG examination of the patient revealed the presence of delayed oscillations in the right cerebral hemisphere, whereas the CSF analysis showed a positive outcome for the 14-3-3 protein. In addition, MRI showed elevated intensities of signal in the frontal, parietal, temporal, and occipital regions, bilaterally. The woman did not have any family history of dementia [68]. One study documented the admission of an elderly patient that had V180I mutation with serious neurological symptoms to the hospital. The patient had intense lesions in the thalamus, right frontal cortex, and temporal cortex. Analysis of brain tissue showed changes in the tissue, empty spaces, scarring, and loss of neurons in most parts of the brain [69]. Another study documented the case of five patients with V180I mutation, The onset of the disease occurred between the ages of 72 and 77 in four of the subjects, while one patient experienced the onset at the age of 57. All five patients exhibiting the V180I mutation displayed either positive or slightly positive results for the presence of 14-3-3 protein in their CSF. The symptoms consisted of cognitive impairment, coordination difficulties, mood disorder, or brain dysfunction [70].

### 5.5 Taiwan

According to the surveillance study conducted by CJDSU from 1998 to 2017, eight cases of gCJD were identified in Taiwan. Of the eight cases identified, two cases consisted of E196A-129 M and one case of R148H-129 M mutation [71]. The age range of the onset of disease was 29–67 years and the mean survival time was 2.5 to 5.5 years [72]. Table 1 summarizes the PRNP mutations that are prevalent in Asian countries.

**Table 1.** **gCJD mutations in Asia**: The table summarizes incidence of gCJD cases against the known mutations in PRNP gene.

Country	Mutation	No. of cases
China	T188K	65
	E200K	41
	E196A	16
	E196K	5
	V203I	3
	R208H	3
	V210I	3
	G114V	2
	R148H	2
	V180I	1
	T183A	1
India	D179N	2
Japan	V180I	211
	M232R	66
	E200K	61
	D179N	5
South Korea	D178N	1
	E200K	2
	M232R	1
	V180I	7
Taiwan	E196A	2
	R148H	1

## 6. Incidence of iatrogenic CJD in Asia

### 6.1 Japan

Iatrogenic cases increased significantly during the 1990s in Asia, mostly due to the transplantation of dura mater grafts which corresponds to 95% of the cases [73]. The CJD surveillance committee of Japan performed a comparative analysis among Japan and other counties and revealed that the prevalence of iCJD was significantly higher than in other counties, the reason being the frequent use of lyodura in 2012 [74]. Most iCJD cases were those of dura mater graft-associated CJD (dCJD) i.e., 0.05% to 0.02% [67,75]. In Japan, 1,241 patients have been identified as definite and probable cases of CJD from 1975 to 2009 among which 77 cases were iCJD [74]. According to Japan’s monitoring system, 2003 showed a significant decline in iCJD cases but in 2017, one case was recorded which was attributed to the long incubation period of this disease [76,77].

Other than dCJD, iCJD can also be caused due to corneal transplant. CJD cases recorded from 1990 to 2006 also included patients who got corneal transplants 1.5 years earlier. But unfortunately, the origin couldn’t be detected as eye banks denied that any donors were infected with prion disease. No patients of iCJD have been reported in Asia who previously received gonadotrophin and human growth hormone (hGH), and this trend can correlate with the careful preparation of human growth hormone through chromatographic purification in Japan [78]. Similarly, since the 1980s no cases of iCJD have been reported to occur via EEG needles and surgical instruments.

### 6.2. Taiwan

A hospital-based nationwide study performed under the Taiwan CJD surveillance unit from 1996 onwards identified 123 patients of CJD but no patients of iCJD [42]. Epidemiological studies in Taiwan indicated that the annual incidence of CJD is lesser compared to other countries. Specifically, iCJD cases are non-existent in Taiwan despite their full-scale tracking system [71,79].

### 6.3. India

Unfortunately, India, like many other countries have not developed any nationwide surveillance unit, hence we are unable to detect the actual number of CJD in general and specifically iCJD patients. Contrary to these induvial studies have shown that there are probable cases of iatrogenic origin in India. For example, a case study conducted from 1971 to 1990 which depicted 3 out of 20 CJD patients had undergone an invasive medical procedure in past [31].

## 7. Comparison to US and Europe

Different countries in Asia exhibit varying rates of sCJD, with certain regions reporting lower incidences compared to others. Among the Asian nations, Japan stands out with the highest reported rates of sCJD. In comparison to numerous Asian nations, the US displays a relatively lower prevalence of sCJD. Approximately 1 out of every 1 million individuals are annually affected by this condition. Significant regional disparities in sCJD surveillance and reporting can be observed in Asia, potentially leading to underreporting in certain areas. The US employs a more centralized and standardized approach to monitor and report rare diseases like sCJD, resulting in more accurate statistical data. The prevalence rates in Europe exhibit substantial variation, with specific Western European countries documenting elevated frequencies of sCJD compared to other Eastern European countries. sCJD has emerged as the central area of interest for multiple extensively financed research networks and institutions throughout Europe, demonstrating noteworthy advancements in the examination and management of this condition [80].

The mutation pattern observed in Japan differed from that observed in the European countries. Among the mutations associated with CJD, the occurrence of V180I (0.2% in the EuroCJD population) and M232R (not detected in the EuroCJD population) was found to be significantly higher in Japan compared to Europe. Among the mutations associated with gCJD, the V180I mutation was found to occur at a rate of 0.2% in the EuroCJD population, while the M232R mutation was not detected in EuroCJD. Interestingly, these mutations were observed to be significantly more common in Japan compared to Europe. On the other hand, the E200K mutation was found to be present in 17.1% of Japanese CJD cases and 41.2% of European cases. Similarly, the V210I mutation was absent in Japanese patients but had a prevalence of 16.2% in European patients. Additionally, the occurrence of the octapeptide insertion was found to be 1.4% in Japan and 9.9% in Europe, while the D178N mutation with either the 129 M/M or V/V genotype was less frequent in Japan compared to European patients [81].

T188K has been documented in two legal instances within the European region, specifically in Austria and Germany. Furthermore, there is a lack of available data regarding the presence of T188K within Asian populations, namely Korean or Japanese, with the exception of Chinese individuals [45]. On the other hand, E196K appears to be more prevalent among European patients, as it has solely been observed in a solitary Chinese case [82]. The occurrence of M129V and E219K showed dissimilarities in East Asia and Europe as well. In the overall population of Koreans and Japanese, a majority of individuals possess the MM allele for M129, whereas the MV allele (approximately 6–7%) and the VV allele (less than 1%) may be infrequent. Conversely, in the general populations of Europe, the MV and VV alleles are rather prevalent (35–51% and 8–12% respectively). Heterozygous E219K was observed in around 7–8% of healthy Koreans and Japanese, while it is likely to be exceedingly rare among the general European population [83].

Studies have shown the frequency of individuals acquiring iCJD after Hgh transplant was higher in European countries (especially in France (119 cases) & UK (65 cases) whereas US reported 29 cases and Asian countries (specifically Japan) reported 77 cases. Analysis of patients with Dura graft transmission showed that Japan comprises of two-third of the cases globally with 83 cases of iCJD being reported till 2005s this may be due to long incubation period. On the other hand, a total of 206 cases were observed in Caucasian population due to transplantation of contaminated Dura graft [84]. Asian countries reported four cases of iCJD cases due to neuronal instruments and corneal transplants whereas no cases were seen in Caucasian population corresponding to former route of exposure, but four deaths were recorded among the patients who have previously undergone corneal transplant [85]. Overall, the frequency of iCJD cases was observed greater in Asian population as compared to Caucasian population.

## 8. Prevalence of other genetic prion diseases

Other Genetic Prion diseases include Gerstmann – Sträussler – Scheinker disease (GSS) and Fatal Familial Insomnia (FFI). GSS can initially present as ataxia or Parkinsonism, with dementia appearing later. The duration of the disease varies, with some patients succumbing within a year and others enduring for over 10 years. Amyloid plaques containing Aβ peptide can be found in the brain, especially the cerebellum [86]. The initial signs of FFI typically involve difficulty sleeping and problems with the autonomic nervous system. As the disease progresses, there are additional challenges with movement and cognition. The duration of the illness is often short, with some patients dying within 2 years of onset. FFI can affect various parts of the nervous system, such as the thalamic nerves, leading to their loss or atrophy. Additionally, there may be PrP^Sc^ deposition in the midbrain or hypothalamus [87]. Prevalence of GSS and FFI cases identified in China, Japan, Korea and India are discussed in this review article.

### 8.1. China

In a study carried out in 2017, five cases of GSS with the P102L mutation were identified. The patients developed the disease at different ages. All cases began with difficulty in walking and progressive ataxia. Affected family members displayed different symptoms. Cognitive decline was common among the patients, but only two had early symptoms. Two individuals had cognitive dysfunction later. The study suggests that unknown genetic or environmental factors may contribute to the phenotypic diversity [88].

In 2017, another case of GSS was identified. The female patient exhibited unstable gait and dysarthria at 44 years old. The patient displayed dysarthria, nystagmus, wide base, and unsteady gait. Brain MRI showed mild diffuse atrophy. The patient’s spinal cord was damaged by herniation [89]. In a study conducted in 2019, 12 GSS patients were identified. Most of these patients exhibited motor dysfunction as an initial symptom [90]. The occurrence of the P105L mutation in Chinese patients is potentially rare, evidenced by the reporting of only a single instance of GSS mutation [91]. FFI with the D178N mutation is also observed frequently in China, with 39% of the analysed cases exhibiting this mutation [92].

### 8.2. Japan

The P102L mutation is prevalent among Japanese patients diagnosed with GSS. The CJD Surveillance Committee has documented the occurrence of this mutation in 39 patients [93]. Familial GSS is the most commonly observed form of the disease, with the age of onset varying among individuals. Additionally, positive cases of the 14-3-3 protein were detected in patients with the P102L mutation. The primary symptoms observed in these patients included spastic paraparesis, gait disturbances, ataxia, and tremor. It is worth noting that not all patients with the P105L mutation exhibited spastic paraparesis. Finally, a recent case involving the P105L mutation was characterized by severe cognitive and gait disturbances, parkinsonism, and swallowing dysfunction [94].

Three cases of the D178N mutation with the MM genotype with FFI were reported. The age of onset ranged from 46 to 57 years, and no PWSC or MRI hyperintensities were observed in these patients. However, at least one case tested positive for 14-3-3 on CSF, and another case involved a 79-year-old patient with D178N and 129 MV genotype who developed sporadic prion disease and tested positive for 14-3-3 on CSF. No abnormalities were seen on MRI or EEG [81]. In another study, a patient with FFI exhibited symptoms such as dysphagia, loss of appetite, abnormal sleep movements, insomnia or hypersomnolence, and later sleep apnoea, as well as tremor, excessive sweating, constipation, impotence or ataxia. MRI showed mild atrophy, but EEG revealed no abnormalities. Histology revealed spongiform changes in the cingulate gyrus and subiculum, gliosis in the thalamus and inferior olivary nucleus, and Western blot analysis indicated a low amount of type 2 PrPSc with the FFI-type glycosylation pattern [95].

### 8.3. Korea

The first cases of GSS in Korea was identified in 2010. The patient was a 46-year-old female that exhibited P102L mutation and displayed symptoms of ataxic gait, language impairment, and cognitive dysfunction. The patient tested positive for the 14-3-3 protein in the CSF and had atypical non-specific slow waves in EEG [96]. P102L was also observed in a patient from another study with definite GSS, but no clinical symptom details were provided by the investigators [97]. In 2019, P102L was reported in another patient, without any family history and the symptoms included gait disturbance, slurred speech, hand clumsiness, and memory dysfunction. MRI showed high signal intensities in the bilateral cortices and mild cerebellar atrophy, while EEG was normal and the CSF tested positive for 14-3-3 protein [98]. A recent report described the first familial case of GSS in Korea. In this family, there was phenotypic heterogeneity. Imaging showed abnormalities in the hemispheric and caudate nuclei. EEG showed mild diffuse slowing and CSF 14-3-3 was positive [99].

Additionally, the first case of FFI was reported in Korea in 2014, the patient was a 34-year-old male that exhibited D178N mutation (homozygous at residue 129) [100]. Another instance of the D178N mutation was described in a 57-year-old male, who was diagnosed with FFI. The patient presented with an irregular sleep-wake cycle, visual hallucinations, myoclonus, ataxic gait, and weight loss. The CSF analysis for 14-3-3 protein yielded negative results, while the EEG indicated widespread slowing without periodic discharge. MRI and PET scans revealed lesions in the white matter and reduced uptake in the bilateral thalamus, respectively [101].

### 8.4. India

In accordance with a study conducted in the year 2020, the initial instance of GSS syndrome was discovered within India, specifically within an Indian household. This family exhibited a notable occurrence of the 305C > T, p.Pro102Leu mutation within the PRNP gene [102].

## 9. Conclusion

The current review attempted to analyse the epidemiological characteristics, clinical examinations, and laboratory features of CJD patients in Asia. sCJD accounts for the highest number of CJD cases out of the three types and is mainly reported in China. gCJD cases have been observed in different ethnicities – T188K, E200 and E196A are most prevalent in China whereas, in Europe, the most dominant mutations are E200K, V210I, and D178N, and in America, the most common mutations are E200K, and D178N [103,104]. China and Japan have differences in PRNP variant profiles, and the most prevalent mutations in Japan (V180I and M232R) are not reported in China. iCJD incidence has declined with the innovation in medical procedures globally as well as in Asia. Epidemiological studies conducted in the Asian region showed the highest incidence of iCJD in Japan. On the other hand, some countries have not reported a single case which corresponds to the lack of surveillance units in those countries.

**Figure 2.**
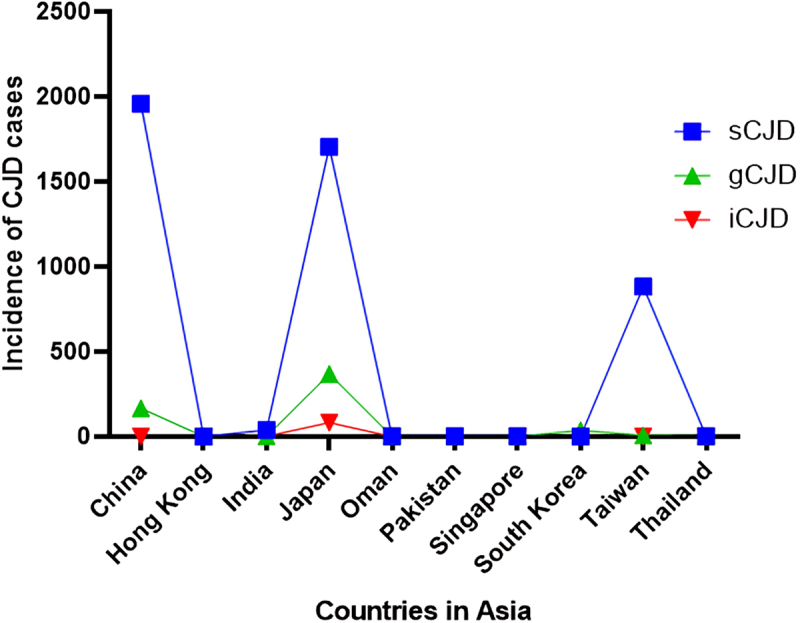
Total cases of CJD reported in Asia from 1986 to 2023. this graph indicates the number of CJD cases reported from the year 1986 to 2023 in Asia. sCJD were the highest among all CJD cases. China (1957) and Japan (1705) reported more cases of sCJD than any Asian country and Hong Kong (1) reported the least. On the other hand, gCJD was highest in Japan (370) and least in India (2). Iatrogenic cases were found to be the least among all CJD types and showed 85 cases overall in Asia, 82 being in Japan and 3 in India.

The current review establishes the presence of all variants of CJD across Asia (Figure 2). However, in most Asian countries in general and Southeast Asian countries in particular, CJD cases are misdiagnosed and often underreported. There is no proper regional surveillance system for CJD. Since Asia is the most populated continent in the world, the actual global prevalence of CJD cannot be estimated until and unless these countries are accounted for. Concrete and reliable surveillance networks are needed across Asia to evaluate the prevalence and incidence of CJD in the region. Ever since we started working on documenting CJD cases in Asia, some cases have been reported in Pakistan with patients exhibiting symptoms of CJD, however, due to a lack of awareness and proper diagnostic measures, the majority remain underdiagnosed and underreported.
